# Black Longevity

**Published:** 1858-02

**Authors:** 


### BLACK LONGEVITY.

THE *London Lancet* thinks it strange that, according to Professor Tucker's Analysis of the United States Census for fifty years, ending in 1840, the chances of living over a hundred years are thirteen to one in favor of the slave. It is ironically said in England, of annuitants and paupers, that they never die; that is, they survive a long time—outlive their generation. The general laws of mind are alike all over the world, whether in hut, or palace, or poor-house. That feeling of certainty, that "*their bread shall be given them, and their water shall be sure*,"

has a marvellously tranquillizing effect on mind and body. Whoever heard of a pauper or a prince being in a hurry? It is the care for to-morrow which eats out the life of the multitude, and sends us all to our graves long before our time, rich and poor together. The literally poor are anxious for *to-morrow's* bread. The already rich are tormented with the fear that somehow or other their wealth may slip out of their hands, and leave them destitute *when they get old.* Many have retired on a fortune, and died from fear of coming to want, and from having nothing to do. Now, whatever may be the disadvantages under which our slaves labor, those disadvantages do not seem to trouble them as much as they do our Abolition friends. We certainly know, from a life-long observation North and South, that there is less vinegar and sadness in a slave's face than in that of his most vociferous friend in the North. We have often gone to hear Abolition stars lecture—masculine, feminine, and neuter; but never saw so much spontaneous gladness in the face of the whole of them, as may be witnessed in any half hour on the Levee, at New Orleans, among the negroes, who are loading and unloading the cotton boats. Hope our Northern subscribers won't bite us for stating these facts; for that they are facts, those who have seen *both* sides, know as well as we do. We are only accounting for the phenomenon, for the benefit of cousin Bull, over the water.

The negroes, then, live longer than the whites, because, as a general thing, slavery don't trouble them much. Secondly, there is no danger of their attaining that bogus elysium, of having nothing to do—an affliction which kills millions of freemen before their time. Thirdly, a slave has no anxiety about his meat and bread. In the first place, he knows he will get it; in the second, if he don't, he believes he has a right to it, and "lives up to his belief." The truth is, the slave is a practical philosopher. He does not grieve and *worry himself to death*, about what he can't help; and in the next place, he believes his master owes him a living, and he feels "bound to have it." Masters fail: slaves never. And if he ever took the trouble to think about it at all, he reasons in this way: "If master's broke, somebody will buy me what a'n't broke, so I can't b'long to poor white folks, no how you can fix it, and this child a'n't a gwine to bodder his self to def' 'bout it."

In short, the chief reasons why our slaves outlive the poorer classes of whites are:

1st. They make themselves pretty well contented with their condition; and,

2d. They have no anxieties about not being provided for.

If, then, our readers wish to live indefinitely long, and in considerable comfort, we advise them to two things:

*First*, Make your calculations to be busy until you die, in doing something useful to yourselves or others; arrange it so that death shall find you with your harness on, manfully battling for humanity and the right. *Second*, Secure a *certain* REGULAR income, however small, which shall be paid to you without any trouble or a peradventure; the nearest approach to such an investment is, *United States six per cents*, IF YOU CAN GET THEM!

